# Beyond Vaccine Efficacy: Redefining the Next Era of Human Papillomavirus Vaccination

**DOI:** 10.3390/vaccines14070633

**Published:** 2026-07-20

**Authors:** Luca Giannella, Andrea Ciavattini

**Affiliations:** Woman’s Health Sciences Department, Gynecologic Section, Polytechnic University of Marche, 60123 Ancona, Italy; andrea.ciavattini@ospedaliriuniti.marche.it

## 1. A New Scientific Era

The greatest challenge in human papillomavirus (HPV) vaccination is no longer scientific. Two decades after the introduction of prophylactic vaccines, overwhelming evidence has established their performance, durable effectiveness, and safety [1,2,3]. Countries with high vaccination coverage have seen substantial reductions in persistent HPV infection, high-grade cervical intraepithelial neoplasia, genital warts, and, more recently, invasive cervical cancer. This has established HPV vaccination as one of the most successful cancer prevention strategies ever implemented [1,2,3].

This remarkable scientific success has transformed the challenges facing researchers and public health authorities. The question is no longer whether HPV vaccines work, it is how their proven effectiveness can be translated into equitable, population-wide protection. Despite the availability of highly effective vaccines and the World Health Organization (WHO)’s strategy to eliminate cervical cancer as a public health problem [4], the disease still causes over 660,000 new cases and about 350,000 deaths each year, with nearly 90% of these occurring in low- and middle-income countries [5]. These disparities reflect persistent inequalities in vaccine access, medical facilities, and program implementation—not biological limitations.

HPV vaccination has entered a new phase. The first era showed that cervical cancer is a preventable disease. The next step is to ensure that prevention reaches every population. Achieving this goal requires more than advances in vaccinology. It demands effective program delivery, equitable access, citizen trust, evidence-based communication, and sustained political commitment [4]. Scientific innovation remains the foundation of HPV prevention, but is no longer sufficient on its own.

## 2. From Evidence to Impact

Very effective vaccines do not stop disease on their own. Their success depends on strong healthcare systems, fair vaccine access, and well-run vaccination programs. As HPV vaccination has grown, research has shifted from demonstrating that vaccines work to maximize their impact. This evolution has prompted several expert groups to advocate renewed efforts to improve vaccine coverage, simplify vaccination strategies, and strengthen implementation programs worldwide [6].

This transition is clear in the growing interest in simplified vaccination schedules. These strategies do more than reduce costs and improve adherence. Single-dose approaches can expand vaccine coverage and speed up progress toward cervical cancer elimination, especially in low- and middle-income countries [1,7]. Umutesi and colleagues showed that a single-dose HPV vaccination program in Kenya could greatly reduce cervical cancer incidence and remain highly cost-effective. This shows how program design can directly influence public health outcomes [8].

Optimizing vaccine delivery is also important in high-income settings. Jørgensen and colleagues showed that catch-up vaccination among women aged 26–30 years significantly reduces the incidence of high-grade cervical lesions. This supports more flexible immunization strategies outside the primary target population [9].

Together, these studies show a fundamental shift in HPV prevention. Scientific innovation has enabled cervical cancer prevention. The next challenge is ensuring these advances are delivered efficiently, equitably, and sustainably in different healthcare settings. In the end, cervical cancer elimination will depend not only on vaccine efficacy but also on the effectiveness of systems that deliver vaccination.

## 3. Trust as a Public Health Intervention

If effective program delivery is the framework foundation of HPV vaccination, public trust is its social foundation. Vaccines prevent disease only when individuals, families, healthcare professionals, and institutions translate scientific evidence into preventive action. Vaccine confidence should no longer be seen merely as a contextual determinant of program performance. It should now be recognized as a public health intervention in its own right [10,11,12].

This evolution marks a broader transformation in public health. The World Health Organization increasingly recognizes behavioral and social drivers as key parts of immunization programs. Scientific evidence alone rarely changes health behavior [12]. Successful vaccination depends on the strength of the evidence and how that evidence is communicated, interpreted, and translated into individual and group decision-making.

The digital environment has quickened this transformation. Health information has never been so widely accessible. At the same time, misinformation spreads rapidly. Giannella and colleagues call the internet a genuine two-edged sword. They highlight the need for healthcare professionals, scientific societies, and public institutions to build authoritative digital presences, not just fight misinformation [13].

Japan’s experience further illustrates this challenge. Takahashi and colleagues reviewed how restoring confidence after years of declining HPV vaccine uptake required more than reassuring safety data. Recovery depended on transparent institutional messaging, coherent public policies, and sustained engagement by healthcare professionals. This shows that trust is built by credible institutions as much as by scientific evidence [14].

Behavioral science offers practical tools for applying these principles in vaccination programs. An intervention in Kosovo, grounded in the Behavior Change Wheel framework, demonstrates how behavioral theory can guide context-specific implementation strategies [15]. Another study in Greece highlights the importance of involving fathers in gender-neutral HPV vaccination programs. This supports a move from mother-centered campaigns to family-centered communication policies [16].

Collectively, these studies illustrate one of the defining lessons of the next era of HPV prevention. Citizen trust should no longer be viewed simply as an outcome of successful vaccination programs but as one of the interventions through which those programs succeed.

## 4. Innovation Beyond Vaccines

Innovation has always propelled progress in HPV prevention. The development of prophylactic virus-like particle (VLP) vaccines changed cervical cancer from a mostly unavoidable consequence of persistent HPV infection to one of the few malignancies that can realistically be prevented by immunization. Today, innovation extends far beyond vaccine design. As current vaccines have proved highly effective, future progress depends on broadening protection, improving affordability, simplifying delivery, and expanding equitable access [17,18].

Among the most promising advances are vaccines targeting the minor capsid protein L2. These approaches exploit conserved epitopes shared across HPV types to deliver broader cross-protection than current L1-based vaccines [17,18]. Two studies in this Special Issue show the swift evolution of this field. Han and colleagues demonstrated broad protection against 17 HPV genotypes using an L2 multimer vaccine in mice. Tsukamoto and colleagues combined L2 immunogens with mRNA technology to induce cross-protective immune responses against both high-risk and low-risk HPV types [19,20].

Innovation also affects vaccine manufacturing and availability. Batool and colleagues explored the expression of HPV-16 and HPV-18 L1 antigens in broccoli. Plant-based production systems could reduce manufacturing costs and improve global access to vaccination [21]. This approach is still experimental, but it illustrates how innovation can address practical barriers to vaccine availability, not just scientific performance.

Innovation in HPV prevention should not be viewed only through a technological lens. Next-generation vaccine platforms, implementation science, behavioral interventions, digital messaging, and health-economic evaluation all serve one aim: translating scientific advances into accessible public health benefits.

## 5. Looking Ahead

The next decade will show whether the extraordinary scientific achievements of HPV vaccination can lead to a fair worldwide impact. The studies in this Special Issue share a central message: eliminating cervical cancer will depend not on a single scientific breakthrough but on the successful integration of vaccine science, program delivery, behavioral research, communication, and health policy [4,22].

Four priorities emerge. First, expanding equitable access to HPV vaccination is essential, especially in the countries most affected by cervical cancer [4,5,23]. Second, program optimization should become a key element of vaccine research, since effective implementation is as important as scientific development [22,24]. Third, strengthening public trust through evidence-based communication and behavioral science must be seen as a core public health intervention, not just a complementary activity [10,11,12,13,14,15,16]. Finally, investment in next-generation vaccine platforms must continue alongside efforts to improve affordability, implementation, and sustainability [17,18,19,20,21].

The first generation of HPV research demonstrated that cervical cancer can be prevented. The next generation must demonstrate that prevention can be delivered equitably, sustainably, and at a global scale. Ultimately, the success of HPV vaccination will be measured not by the sophistication of future vaccines but by health systems’ ability to ensure that their benefits reach every population. The future of HPV vaccination will depend not only on developing better vaccines but on building better vaccination systems.

**Figure 1:**
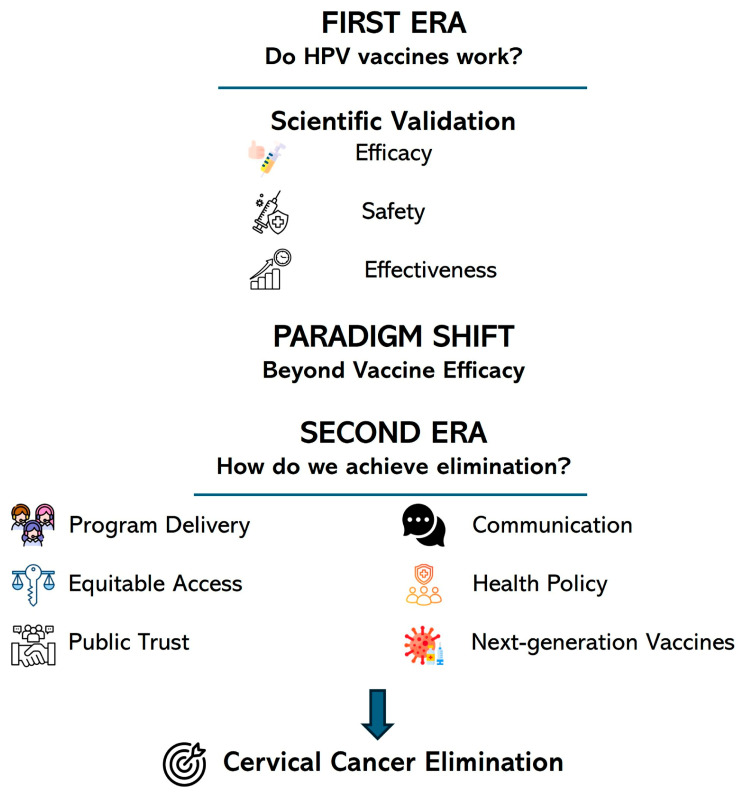
**Beyond vaccine efficacy: a conceptual framework for the next era of HPV vaccination.** The first era of HPV vaccination established the efficacy, safety, and real-world effectiveness of prophylactic vaccines. The next era extends beyond scientific validation and focuses on integrating program delivery, equitable access, public trust, behavioral science, communication, health policy, and next-generation vaccine innovation to achieve the World Health Organization’s goal of eliminating cervical cancer. Icons were obtained from https://www.flaticon.com.

Figure 1 summarizes the conceptual framework proposed in this Editorial, illustrating the transition from demonstrating vaccine efficacy to integrating the scientific, organizational, behavioral, and policy dimensions required to achieve cervical cancer elimination.

## References

[1] World Health Organization (2022). Human papillomavirus vaccines: WHO position paper. Wkly. Epidemiol. Rec..

[2] Drolet M., Bénard É., Pérez N., Brisson M. (2019). HPV Vaccination Impact Study Group. Population-level impact and herd effects following the introduction of human papillomavirus vaccination programmes: Updated systematic review and meta-analysis. Lancet.

[3] Falcaro M., Castañon A., Ndlela B., Checchi M., Soldan K., Lopez-Bernal J., Elliss-Brookes L., Sasieni P. (2021). The effects of the national HPV vaccination programme in England, UK, on cervical cancer and grade 3 cervical intraepithelial neoplasia incidence: A register-based observational study. Lancet.

[4] World Health Organization (2020). Global Strategy to Accelerate the Elimination of Cervical Cancer as a Public Health Problem.

[5] Bray F., Laversanne M., Sung H., Ferlay J., Siegel R.L., Soerjomataram I., Jemal A. (2024). Global cancer statistics 2022: GLOBOCAN estimates of incidence and mortality worldwide for 36 cancers in 185 countries. CA Cancer J. Clin..

[6] Bogani G., Ghelardi A., Sopracordevole F., Annoni M., Ciavattini A., Giannella L., De Vincenzo R., Cattani P., Barbero M., Vercellini P. (2023). Human papillomavirus (HPV) vaccination: A call for action in Italy. Int. J. Gynecol. Cancer.

[7] Barnabas R.V., Brown E.R., Onono M.A., Bukusi E.A., Njoroge B., Winer R.L., Galloway D.A., Pinder L.F., Donnell D., Wakhungu I. (2022). Efficacy of Single-Dose Human Papillomavirus Vaccination among Young African Women. NEJM Evid..

[8] Umutesi G., Hathaway C.L., Heitner J., Jackson R., Miano C.W., Mugambi W., Khalayi L., Mwenda V., Oluoch L., Nyangasi M. (2024). The Potential Impact of a Single-Dose HPV Vaccination Schedule on Cervical Cancer Outcomes in Kenya: A Mathematical Modelling and Health Economic Analysis. Vaccines.

[9] Jørgensen A.S., Simonsen G.S., Sørbye S.W. (2025). Impact of HPV Catch-Up Vaccination on High-Grade Cervical Lesions (CIN2+) Among Women Aged 26–30 in Northern Norway. Vaccines.

[10] Larson H.J., Jarrett C., Eckersberger E., Smith D.M., Paterson P. (2014). Understanding vaccine hesitancy around vaccines and vaccination from a global perspective: A systematic review of published literature, 2007–2012. Vaccine.

[11] Brewer N.T., Chapman G.B., Rothman A.J., Leask J., Kempe A. (2017). Increasing Vaccination: Putting Psychological Science Into Action. Psychol. Sci. Public Interest.

[12] World Health Organization (2022). Behavioural and Social Drivers of Vaccination: Tools and Practical Guidance for Achieving High Uptake.

[13] Giannella L., Grelloni C., Natalini L., Sartini G., Lavezzo F., Cicoli C., Bernardi M., Bordini M., Petrini M., Petrucci J. (2025). The Role of Internet Information on Anti-HPV Vaccines: A Comprehensive Overview of a Double-Edged Sword. Vaccines.

[14] Takahashi T., Ichimiya M., Tomono M., Minoura R., Kinoshita T., Imanishi Y., Sakamoto M., Mitsunami M., Song M., Inaba K. (2025). Overcoming HPV Vaccine Hesitancy in Japan: A Narrative Review of Safety Evidence, Risk Communication, and Policy Approaches. Vaccines.

[15] Miftari Basholli F., Haxhiu E., Humolli I., Berisha M., Nielsen S.M., Warsi S.K. (2025). Rapid Development of a Theory-Based Targeted Intervention and Communication Plan for HPV Vaccine Introduction in Kosovo Using the Behaviour Change Wheel Model. Vaccines.

[16] Christodoulou M., Paraforou C., Rouka E., Toska A., Papagiannis D. (2026). Are Fathers Being Left Behind? Gender Differences in Parental HPV Vaccination Knowledge and Attitudes Toward Sons’ Vaccination in Greece. Vaccines.

[17] Wang R., Huang H., Yu C., Li X., Wang Y., Xie L. (2024). Current status and future directions for the development of human papillomavirus vaccines. Front Immunol..

[18] Amiri S., Rasekh S., Moezzi S.M.I., Seifi N., Fatemi S.A., Fathi S., Bagheri A., Negahdaripour M. (2025). Prophylactic vaccines against HPV-caused cervical cancer: Novel vaccines are still demanded. Infect. Agent Cancer.

[19] Han Z., Wang S., Mu T., Zhao P., Song L., Zhang Y., Zhao J., Yin W., Wu Y., Wang H. (2024). Vaccination with a Human Papillomavirus L2 Multimer Provides Broad Protection against Seventeen Human Papillomavirus Types in the Mouse Cervicovaginal Challenge Model. Vaccines.

[20] Tsukamoto K., Yamashita A., Maeki M., Tokeshi M., Imai H., Fukao A., Fujiwara T., Okudera K., Mizuki N., Okuda K. (2024). Enhanced Broad-Spectrum Efficacy of an L2-Based mRNA Vaccine Targeting HPV Types 6, 11, 16, 18, with Cross-Protection Against Multiple Additional High-Risk Types. Vaccines.

[21] Batool N., Ahsan K., Qadeer K., Fajar A., Farid A., Sameeullah M., Ijaz F., Malik M.S., Tariq F.A., Lössl A.G. (2026). In Silico Design and Subsequent Expression of HPV-16 and -18 L1 Vaccine Antigens in Broccoli. Vaccines.

[22] Arbyn M., Gultekin M., Morice P., Nieminen P., Cruickshank M., Poortmans P., Kelly D., Poljak M., Bergeron C., Ritchie D. (2021). The European response to the WHO call to eliminate cervical cancer as a public health problem. Int. J. Cancer.

[23] Brisson M., Kim J.J., Canfell K., Drolet M., Gingras G., Burger E.A., Martin D., Simms K.T., Bénard É., Boily M.C. (2020). Impact of HPV vaccination and cervical screening on cervical cancer elimination: A comparative modelling analysis in 78 low-income and lower-middle-income countries. Lancet.

[24] World Health Organization (2023). Framework for Monitoring the Implementation of the WHO Global Strategy to Accelerate the Elimination of Cervical Cancer as a Public Health Problem.

